# Study of the Interference between *Plectranthus* Species Essential Oils from Brazil and Aminoglycosides

**DOI:** 10.1155/2013/724161

**Published:** 2013-04-04

**Authors:** Fabíola Fernandes Galvão Rodrigues, José Galberto Martins Costa, Fábio Fernandes Galvao Rodrigues, Adriana Rolim Campos

**Affiliations:** ^1^Programa de Pós-Graduação em Biotecnologia, Rede Nordeste de Biotecnologia, Universidade Estadual do Ceará, Avenida Paranjana 1700, Campus do Itaperi, 60740-000 Fortaleza, CE, Brazil; ^2^Programa de Pós-Graduação em Bioprospecção Molecular, Departamento de Química Biológica, Laboratório de Pesquisa de Produtos Naturais, Universidade Regional do Cariri, Rua Cel. Antônio Luiz 1161, Pimenta, 63105-000 Crato, CE, Brazil; ^3^Programa de Pós-Graduação em Biotecnologia, Rede Nordeste de Biotecnologia, Universidade de Fortaleza, Avenida Washington Soares 1321, Edson Queiroz, 60811-905 Fortaleza, CE, Brazil

## Abstract

*Plectranthus* is one of the most representative genera of Lamiaceae family. In this study, the essential oils from *Plectranthus amboinicus*, *Plectranthus ornatus*, and *Plectranthus barbatus* were investigated for their chemical composition and antimicrobial and modulatory activities. The major components found were carvacrol (54.4%—*P. amboinicus*) and eugenol (22.9%—*P. ornatus* e 25.1%—*P. barbatus*). *In vitro* antimicrobial activity was conducted against *Escherichia coli*, *Proteus vulgaris*, *Bacillus cereus*, *Pseudomonas aeruginosa*, *Staphylococcus aureus*, and *Staphylococcus aureus* (multiresistant) using microdilution method. The results of bioassay showed that all strains were sensitive to the oils, except *P. aeruginosa* that was resistant to *P. amboinicus* and *P. ornatus*. A synergistic effect of all essential oils combined with the aminoglycosides was demonstrated. These results show that *P. amboinicus*, *P. ornatus*, and *P. barbatus* inhibit the growth of pathogenic microorganism, and besides this they present antibiotic modifying activity, providing a new perspective against the problem of bacterial resistance to antibiotics.

## 1. Introduction

The increasing antibiotic resistance has improved the interest in the development of new antimicrobial compounds from medicinal plants. The need for new classes of safe and natural antimicrobials able to control the microorganism growth and infections has become more urgent [1]. Synthetic drugs have been associated with several side effects on human health. Furthermore, microorganisms indicated a resistance to synthetic antimicrobial agents, which is a serious and immediate concern. Taken together, these facts point to the need for new alternative drugs from natural source.

However, the identification and evaluation of these products, presenting optimized levels of antimicrobial activity, can be considered as an important world challenge for food and drugs industries.

Essential oils or some of their components are used in perfumes and makeup products, in sanitary products, in dentistry, in agriculture, as food preservers and additives, and as natural remedies [2]. This range of uses is attributed to the essential oils chemical composition, mainly terpenes and terpenoids. Uncountable studies point to the antimicrobial effect of essential oils and isolate constituents, and besides this interactions between the different natural chemical components and synthetic antibiotics can occur, leading to a potentialization of the modulatory effect [3, 4].

Analysis of the antimicrobial activity of chemical constituents of the oils, alone or in combination, in different treatments using different microorganisms can produce lethal effects at lower doses, avoiding changes and providing various perspectives on the problem of bacterial resistance to antibiotics [1, 5].

Some synthetic chemical compounds or from natural sources are able to increase the activity of specific antibiotics and to revert the natural resistance of particular bacteria against some antibiotics [6, 7]. The ability of natural or nonconventional synthetic antibiotics to reinforce the antibiotic activity or to modify the resistance allows the classification of these compound as modifiers of antibiotic activity [8].

Genus *Plectranthus* (Lamiaceae) comprises about 300 species of herbs and shrubs native to tropical regions. The most frequently cited use of species of *Plectranthus* is for their medicinal properties, which accounts for over 85% of all uses [9, 10]. The species are used for the treatment digestive disturbances (21 species), skin affections (20 species), respiratory infections (15 species), general infections and fever (20 species), genitourinary infections (08 species), pain (09 species), musculoskeletal conditions (09 species). *Plectranthus *species are used also to treat blood and circulation conditions and the nervous system disturbances [10].

It is known that *Plectranthus* species present a great biosynthetic capacity to produce diverse chemical classes from secondary cell metabolism, mainly diterpenoids and triterpenoids, some of them showing confirmed biological properties [11].

*Plectranthus amboinicus*, popularly known as “malvarisco,” “hortelã grande” and “hortelã-da-folha-grossa,” is a long lengh, perennial, aromatic herb. This specie is one of the most popular medicinal plants used in the northeast region of Brazil because its antimicrobial, balsamic, and anti-inflammatory properties [12, 13]. Previous studies have demonstrated that the leaves of this specie possess bronchodilator, antituberculous, antibacterial, antifungal, and antiviral activities [13–16].

*Plectranthus barbatus*, known in popular medicine as “malva-santa” or “malva-sete-dores,” is a herbaceous subshrub presenting aromatic, bitter and flexible leaves. The interest in studying this specie was stimulated by the wide use of its leaves to treat digestive disturbances in substitution for “boldo do Chile” (*Peumus boldus*). *P. barbatus* is also emplyoed in the treatment of nauseas, gastritis, and intestinal spasms [10]. Pharmacological studies have reported the gastric hyposecretive activity of *P. barbatus. *This activity is probably related to the presence of the diterpene barbatusin that possesses antidyspeptic and antiulcerous properties [10].

*Plectranthus ornatus *(“boldo gambá” and “boldo miúdo”) is largely employed in popular medicine to treat hepatic and digestive affections. This pharmacological importance is attributed to the diterpenes found in its leaves [17]. Besides barbatusin previously found in *P. barbatus*, a new diterpene in *P. ornatus* leaves was reported by Albuquerque et al. [18, 19]. The authors propose the substitution of *P. ornatus *by *P. barbatus, *even without the confirmation of the gastric hyposecretive activity. Rijo et al. [20] showed the isolation of 3 diterpenes, similarly to forskolin, in the leaves of *P. ornatus* and two of them possess antibacterial activity.

Here we describe the first report of the antibiotic modifier activity of *Plectranthus* essential oils.

## 2. Materials and Methods

### 2.1. Plant Material

The aerial parts of *P. amboinicus*, *P. barbatus*, and *P. ornatus* were collected in March 2010, from the Medicinal Plants Garden at the Regional University of Cariri (URCA). Voucher specimens were deposited in the Herbarium Caririense D*α*rdano de Andrade Lima (numbers 3037, 3038, and 3039, resp.). 

### 2.2. Essential Oil Extraction

Samples of *P. amboinicus*, *P. barbatus,* or *P. ornatus* fresh leaves (500 g) were triturated and submitted to hydrodistillation process, in a Clevenger-type apparatus for 2 hours. The collected essential oils were subsequently dried by anhydrous sodium sulfate (Na_2_SO_4_) and stored under refrigeration at <4°C until analyzed and tested.

### 2.3. GC/MS Analysis

Analysis of the volatile constituents of essential oil was carried out on a Hewlett-Packard Model 5971 GC/MS using a nonpolar DB-5 fused silica capillary column (30 m × 0.25 mm i.d., 0.25 *μ*m film thickness) carrier gas helium, flow rate 1 mL/min, and with split mode. The injector temperature and detector temperature were 250°C and 200°C, respectively. The column temperature was programmed from 35°C to 180°C at 4°C/min, and then 180°C to 250°C at 10°C/min, split ratio (1 : 30); mass spectra were recorded from 30 to 450 m/z, electronic impact 70 eV. Individual components were identified by computer library MS searches using retention indices as a preselection routine and visual inspection of the mass spectra from the literature for confirmation.

### 2.4. Antibacterial Activity and Minimal Inhibitory Concentration (MIC)

The antibacterial activities of the essential oils were investigated by employing a microdilution method, recommended by M7-A6 [21]. The assay was carried out with five bacterial species obtained from Fundação Oswaldo Cruz (FIOCRUZ): *Escherichia coli* ATCC 25922, *Proteus vulgaris* ATCC 13135, *Bacillus cereus* ATCC 33018, *Pseudomonas aeruginosa* ATCC 15442, *Staphylococcus aureus* ATCC 12692, and a multiresistant strain *Staphylococcus aureus* SA 358. The initial solution of essential oils was diluted to reach a final concentration in the range of 512 to 8 *μ*g/mL. All experiments were performed in triplicate and the microdilution trays were incubated at 35 ± 2°C for 24 h. Antibacterial activity was detected using a colorimetric method by adding 25 *µ*L of resazurin staining (0.01%) aqueous solution in each well at the end of the incubation period. The minimal inhibitory concentration (MIC) was defined as the lowest EOLm concentration able to inhibit the bacteria growth, as indicated by resazurin staining (bacteria died cells are not able to change the staining color by visual observation, blue to red).

### 2.5. Modulatory Activity by Direct Contact

To evaluate the essential oils as modulators of antibiotic resistance, the MICs of aminoglycosides (kanamycin, amikacin, and gentamicin) against the analyzed strains were determined in the presence or absence of the essential oils using the microdilution test. Subinhibitory concentrations (MIC 1/8) in 10% BHI were used.

The antibiotics solutions (1000 *μ*g/mL) were prepared in distillated water for use the same day. A total of 100 *μ*L of the antibiotic solution, using use serial dilutions (1 : 2), was added to the wells containing 10% BHI and the diluted bacterial suspension (1 : 10). Microplates were incubated for 24 h at room temperature and the antibacterial activity was determined as described before.

## 3. Results and Discussion

### 3.1. Essential Oils Yields and Composition

The essential oils obtained by hydrodistillation presented different yields: 0.3% *P. amboinicus*, 0.1% *P. barbatus,* and 0.2% *P. ornatus*, v/v. According to Table 1, the main constituents found were carvacrol (54.4%—*P. amboinicus*) and eugenol (22.9%—*P. ornatus* and 25.1%—*P. barbatus*). Although these species belong to the same genus, they exhibit distinct chemical and pharmacological properties [18]. **α**-humulene, **β*-*caryophyllene, carvacrol, thymol, *cis*-**β**-ocimene, and *α*-pinene were also found as uncommon constituents.

As reported by de Albuquerque et al. [18, 19], the essential oil from *P. ornatus* presented *trans*-*β*-caryophyllene (9.6%–62.4%), eugenol (38.0%), and timol (14.1%) as the major components.

Previous works show that the essential oils from aerial parts and roots from *P. barbatus* possess *α*-pinene, *β*-phellandrene, (Z)-*β*-ocimene, manol, and abietadiene as main constituents [18, 22].

Costa [23] identified 23 components (94.13%) in the essential oil from *P. barbatus* leaves, with predominance of sesquiterpenes (10) and monoterpenes (09). Two oxygenated sesquiterpenes and two diterpenes were found too. The essential oil from fresh leaves of *P. amboinicus* collected in the South of Ceará is composed of timol (64.3%), *p*-cymene (10.3%), *γ*-terpinene (9.9%) and *β*-caryophyllene (2.8%) [14].

Twenty-six compounds were found in an Indian sample of *P. amboinicus* essential oil. The major chemical compounds were carvacrol (28.65%) followed by thymol (21.66%), *α*-humulene (9.67%), undecanal (8.29%), *γ*-terpinene (7.76%), **ρ**-cymene (6.46%), caryophyllene oxide (5.85%), **α**-terpineol (3.28%), and *β*-selinene (2.01%) [24].

### 3.2. Antimicrobial Activity

The antibacterial activities of *Plectranthus* essential oils were assayed *in vitro* by a microdilution method against six pathogenic bacteria.  Table 2 summarizes the microbial growth inhibition by each essential oil. According to the results, the essential oils were found to be active against Gram-positive and Gram-negative strains.

*P. amboinicus* essential oil was found to be active against all pathogenic bacteria except *Pseudomonas aeruginosa*. The strongest antibacterial activity was seen against *Staphylococcus aureus *SA 358 with a MIC value of 32 *µ*g/mL followed by *Proteus vulgaris* ATCC 13315 MIC 64 *µ*g/mL. Costa et al. [14] reported that the essential oil from *P. amboinicus*, using the disc diffusion assay, inhibited the growth of *Staphylococcus aureus* ATCC 10390, *Proteus vulgaris* ATCC 13315, and *Aeromonas caviae* ATCC 15468. The essential oil was not active against *Escherichia coli* ATCC 25922, and *Enterobacter cloacal* ATCC 23355. The extract of *P. amboinicus* was active against seven *Staphylococcus* strains (halo inhibition of 13 mm), but it was not active against *P. aeruginosa* and *Candida *ssp. [25]. Costa et al. [14] evaluated the essential oil from *Z. articulatum* against a multiresistant *S. aureus* (SA 358) and a MIC value of *µ*g/mL was found; this value (<1000 *µ*g/mL) was clinically significant. So, the results obtained here are considered relevant, once a lower MIC value was found against the same multiresistant strain [26]. 

*P. barbatus* was active against all tested strains, mainly against the Gram-positive *Bacillus cereus* ATCC 33018 and* Staphylococcus aureus *ATCC  12692 with MIC values of 64 and 32 *µ*g/mL, respectively.

Previous studies showed that roots, barks, and leaves of *Plectranthus barbatus*, collected in the south region of Africa, exhibited antibacterial activity. This confirms that Lamiaceae species are recognized by the presence of terpenoids with antifungal, antibacterial, and insecticidal actions [27]. Matu and Staden [27] verified that hexane and methanolic extracts of roots, barks, and leaves from *P. barbatus* were active against *Bacillus subtilis*, *Micrococcus luteus,* and *Staphylococcus aureus*.

*P. ornatus* was also active against all strains tested, except *P. aeruginosa*. The lower MIC values found was 128 *µ*g/mL, for *Proteus vulgaris* and *Staphylococcus aureus *ATCC 12692.

Essential oils, rich in phenolic compounds, possess high levels of antimicrobial activity [28–30], like the results obtained here.

The phenolic compound, thymol and carvacrol, have been described as the major components of essential oils from *Plectranthus* species, being responsible for the antimicrobial properties of the oils [31]. Carvacrol and thymol present capability of dissolving into the cytoplasm membrane aligning among the fatty acid chains providing an increase in the cytoplasm membrane passive permeability [32, 33].

Ait-Ouazzou et al. [3] showed that *P. aeruginosa *was the bacterial strain most resistant to several essential oil constituents, being only moderately inhibited by carvacrol. Therefore, oxygenated monoterpenes showed higher inhibitory activity on microbial growth than did hydrocarbons. Phenolic compounds, as carvacrol and timol, are more efficient than hydrocarbons, alcohols, and esters.

The bacteriostatic properties of essential oils rich in thymol were previously reported [34, 35]. Thymol presented a better activity against *P. aeruginosa*, *S. aureus* and *E. coli *than did an essential oil rich in thymol [36]. 

Several studies have demonstrated that essential oils and extracts from other *Plectranthus* species are between the most potent antimicrobial natural products [27, 37, 38]. In this study, it was shown that all essential oils tested were more effective against Gram-positive strains, in comparison to Gram-negative. These results are in agreement with the report of Othman (2005) [39]; in this study Gram-negative microorganisms are more resistant to antimicrobials agents. This resistance may be is related to the presence of a peptidoglycan barrier in Gram-negative strains, thus restricting of antibiotic access to the target.

### 3.3. Modulatory Activity of Direct Contact Assay

Table 3 shows that antibiotic activity of amikacin, kanamycin and gentamicin was better in the presence of the essential oils. *P. amboinicus* potentialized the antibiotic activity of all drugs against all strains tested. *P. ornatus* did not interfere with the amikacin effect against *E. coli* (ATCC 25922). The same response was observed with gentamicin against *B. cereus* (ATCC 33018). A synergistic effect between *P. barbatus* and aminoglycosides was observed.

Essential oils may interact with and affect the plasma membrane, interfering with respiratory chain activity and energy production [8, 40]. The mechanisms by which essential oils can inhibit microorganisms involve different modes of action and in part may be due to their hydrophobicity [41]. Some research works show that essential oils (*Lantana camara, Melaleuca leucadendron, and Ocimum gratissimum*) demonstrated synergism with antibiotics by direct contact [42].

Components of essential oils as thymol and carvacrol may act as membrane permeabilizers, enhancing the intake of antibiotics [43]. Impairment of bacterial enzyme systems may also be a potential mechanism of action [44]. 

In this way, this report represents the first report regarding the modulatory activity of essential oils from *Plectranthus *species. Others works indicated different antibiotics combinations tested *in vitro *and clinically applied; however, combinations between natural products and synthetic drugs have been less published [45]. The results obtained here suggest that the essential oils from *Plectranthus* species can suppress the growth of Gram-positive and Gram-negative bacteria and they could be a source of metabolites with antibacterial modifying activity to be used as adjuvants to antibiotic therapy against these pathogens. So research should be stimulated to identify more natural compounds with synergistic behavior.

## 4. Conclusion

The results of this study suggest that the chemical components of essential oils of the *Plectranthus *genus can suppress the growth of Gram-positive and Gram-negative pathogenic bacteria. Especially for large amount of phenolic compounds such as thymol, carvacrol, and eugenol, this can modify the activity of antibiotics and therefore be used as adjuncts to antibiotic therapy against these pathogens. Thus, the search for natural compounds with antimicrobial activity and behavior is necessary for synergistic emergency control and treatment of diseases.

## Figures and Tables

**Table 1:** Chemical composition (%) of the leaf oils of *P. amboinicus*, *P. ornatus*, and *P. barbatus*.

Components	IR^a^	IR^b^	Composition (%)		
			*P. amboinicus *	*P. ornatus *	*P. barbatus *
*α*-pinene	941	939	0.8	0.7	0.3
sabinene	972	975	—	1.2	0.6
**β**-pinene	981	979	1.2	2.2	—
**β**-myrcene	987	991	—	—	1.8
*p*-cymene	1022	1025	10.3	—	—
*cis*-**β**-ocimene	1032	1037	1.3	2.2	1.9
*trans*-**β**-ocimene	1046	1050	—	0.9	1.2
*γ*-terpinene	1064	1060	5.9	—	—
Terpinen-4-ol	1173	1177	0.9	2.5	—
Thymol methyl ether	1235	1235	1.6	—	—
Thymol	1288	1290	1.3	16.6	15.3
Carvacrol	1296	1299	67.9	13.9	12.1
Eugenol	1365	1359	—	22.9	25.1
**α**-copaene	1375	1377	—	2.4	8.9
**β**-cubebene	1385	1388	—	1.2	3.7
**β*-*caryophyllene	1422	1419	2.8	18.2	10.7
*(E)-*α*-*bergamotene	1430	1435	1.4	—	0.5
**α**-humulene	1451	1455	0.7	0.8	1.6
Aromadendrene	1458	1460	1.2	—	—
**β**-bisabolene	1509	1506	—	—	—
**δ**-cadinene	1519	1523	—	—	1.4
*trans*-nerolidol	1558	1563	—	0.8	0.2
Caryophyllene oxide	1582	1583	—	0.3	2.4
epi-**α**-cadinol	1638	1640	—	—	0.6

Total identified			97.3	86.4	88.3

^a^Relative retention indices experimental: n-alkanes were used as reference points in the calculation of relative retention indices. ^b^Relative retention indices (the literature values).

**Table 2:** Minimal inhibitory concentration (MIC) of *P. amboinicus*, *P. ornatus*, and *P. barbatus* fresh leaves essential oils.

Microorganism	MIC (*µ*g/mL)		
	*P. amboinicus *	*P. ornatus *	*P. barbatus *
*E. coli *ATCC 25922	256	512	512
*P. vulgaris *ATCC 13315	64	128	256
*B. cereus *ATCC 33018	512	512	64
*P. aeruginosa *ATCC 15442	≥1024	≥1024	512
*S. aureus *ATCC 12692	128	128	32
*S. aureus *SA 358	32	256	512

**Table 3:** MIC values of aminoglycosides in the presence and absence of essential oils.

	Strains		Amikacin (*µ*g/mL)	Kanamycin (*µ*g/mL)	Gentamicin (*µ*g/mL)
*P. amboinicus *	*S*. *a*. 12692	MIC	64	64	128
		EO (16 *µ*g/mL)	32	16	64
	*S*. *a*. 12624	MIC	128	64	64
		EO (4 *µ*g/mL)	64	8	16
	*B*. *c*. 33018	MIC	128	64	64
		EO (64 *µ*g/mL)	16	32	16
	*P*. *v*. 13315	MIC	64	32	128
		EO (8 *µ*g/mL)	16	8	16
	*E*. *c*. 25922	MIC	64	128	64
		EO (32 *µ*g/mL)	32	64	32

*P. ornatus *	*S*. *a*. 12692	MIC	64	64	128
		EO (64 *µ*g/mL)	16	8	8
	*S*. *a*. 12624	MIC	128	64	64
		EO (64 *µ*g/mL)	64	32	32
	*B*. *c*. 33018	MIC	128	64	64
		EO (16 *µ*g/mL)	16	32	64
	*P*. *v*. 13315	MIC	64	32	128
		EO (16 *µ*g/mL)	32	16	32
	*E*. *c*. 25922	MIC	64	128	64
		EO (32 *µ*g/mL)	64	16	8

*P. barbatus *	*S*. *a*. 12692	MIC	64	64	128
		EO (64 *µ*g/mL)	16	8	8
	*S*. *a*. 12624	MIC	128	64	128
		EO (64 *µ*g/mL)	16	32	32
	*B*. *c*. 33018	MIC	128	64	64
		EO (16 *µ*g/mL)	64	32	16
	*P*. *v*. 13315	MIC	64	32	128
		EO (16 *µ*g/mL)	32	16	32
	*E*. *c*. 25922	MIC	64	128	64
		EO (32 *µ*g/mL)	8	32	16

*E. c. (Escherichia coli), P. v. (Proteus vulgaris), B. c. (Bacillus cereus), P. a. (Pseudomonas aeruginosa).*
